# Hepatitis B knowledge among women of childbearing age in three slums in Mumbai: a cross-sectional survey

**DOI:** 10.1186/s41124-016-0007-7

**Published:** 2016-05-12

**Authors:** Swati Jha, Divyesh Devaliya, Susan Bergson, Shripad Desai

**Affiliations:** 1 AmeriCares india Foundation, Mumbai, India; 2 AmeriCares, Stamford, CT USA

**Keywords:** Hepatitis B, Pregnant women, Women of child bearing age, Hepatitis B testing, Hepatitis B transmission

## Abstract

**Background:**

More than 17 million people in India are chronically infected with the hepatitis B virus (HBV). Approximately one million of the 26 million children born in India annually will develop chronic HBV infection in the course of their lives. Studies have put the HBsAg prevalence rate among pregnant women in India between 0.9 % and 3.1, indicating a considerable need for public health interventions aimed at protecting their offspring from infection. The PAHAL project in Mumbai, India, conducted an HBV knowledge survey among women of childbearing age in three local slum communities preparatory to planning a comprehensive HBV education intervention targeting this population.

**Methods:**

Female health workers approached all households in three designated slum neighborhoods: one each in the eastern suburbs (ES), western suburbs (WS) and Island City (IC). Female residents aged 18–45 were invited to participate in the study, and those who agreed to participate responded to a questionnaire that was administered in the form of an oral interview. The five sections of the questionnaire addressed demography, hepatitis B knowledge, personal risk related to hepatitis B, pregnancy and childbearing history, and the participant’s most recent pregnancy. A descriptive statistical analysis was performed.

**Results:**

Health workers submitted 6571 interview forms that were suitable for analysis (ES, 28 %; WS, 34 %; IC, 38 %). Large proportions of study participants were married (89 %), were not employed (94 %) and had completed less than 12 years of school (87 %). Only 240 (3.6 %) women answered yes when asked if they knew about hepatitis B. Among those women, there were high levels of accurate knowledge regarding some modes of hepatitis B transmission but low levels of accurate knowledge regarding other modes. Twenty-two percent of 739 women who had given birth within the previous 36 months reported that they had been tested for HBV during pregnancy. While 70 % of these women reported that their children had received three doses of hepatitis B vaccine, reported vaccination levels varied greatly across the three study areas.

**Conclusions:**

Despite the availability of a safe HBV vaccine, which is free for newborns and infants in many parts of India, preventing mother-to-child transmission of HBV remains a major challenge. Low awareness of HBV among women of childbearing age in Mumbai-area slums indicates a need for educational interventions targeting this population.

## Background

An estimated 240 million people globally are chronically infected with the hepatitis B virus (HBV), with a great deal of geographical variation in disease prevalence [1]. The World Health Organization (WHO) has reported that India's chronic HBV prevalence level is between 3 % and 4.2 % [2], while a recent systematic review found a lower prevalence level of approximately 1.5 % [3]. India appears to have lower chronic HBV prevalence than many countries in Asia and the Pacific, but the sheer size of the population translates into a large disease burden. To put the numbers in perspective, the 1.5 % prevalence estimate noted in the systematic review represents an infected population of more than 17 million [3].

It is estimated that approximately one million of the 26 million children born in India annually will develop chronic HBV infection in the course of their lives [4]. The natural history of the disease is such that acquiring HBV at birth or in childhood results in a higher chance of developing chronic infection compared to acquiring it in adulthood [1]. Based on the similar prevalence of infection in different age groups in the under-age-five population, a study in the outpatient department of a large hospital in India concluded that most of the hepatitis B cases encountered in the subjects were the result of vertical transmission [5]. Studies from different parts of India have put the HBsAg prevalence rate among pregnant women between 0.9 % and 3.1 % [6–8], suggesting that there is a considerable need for public health interventions aimed at protecting their offspring from infection.

Universal vaccination with screening of pregnant women plus the administration of hepatitis B immunoglobulin to the children of HBV-infected women at birth is the most effective strategy for reducing, and ultimately, eradicating hepatitis B, but insufficient financial resources and health system constraints prevent some countries from fully implementing this strategy. India introduced universal immunization against hepatitis B in 10 states in the year 2002, and in 2011, scaled up this intervention to target the entire country. WHO and UNICEF estimate that India had 8 % national coverage of hepatitis B birth-dose immunization in 2011, and that national coverage of the three-dose vaccine was 44 % in the same year [9]. Although pregnant women in India can undergo free HBV testing at government testing sites in major cities, those who learn that they have HBV may be unable to take measures to protect their newborns from infection as a result of financial barriers or other reasons.

In this context, AmeriCares India and United Way India collaborated to establish the PAHAL project to provide HBV prevention education in three slum communities in the Mumbai metropolitan area. The word *pahal,* which means “new beginning” in Hindi, was chosen because the project sought to promote community awareness of a disease that has received little attention from the Indian government and Indian medical community despite its heavy impact.

Slums in Mumbai are typically built illegally on government land, and sometimes on private land, and are characterized by a shortage of living space and inadequate water and sanitation facilities. PAHAL was planned as a comprehensive educational intervention to promote targeted hepatitis B screening, hepatitis B immunization, medical care and related interventions among women of childbearing age in slum communities, particularly pregnant women. Before the intervention, a survey was undertaken to determine what these populations already knew about hepatitis B. PAHAL planners also sought to establish whether women of childbearing age in the three targeted communities had similar or different needs in relation to HBV prevention education. This paper reports on the baseline survey that was conducted to inform decision-making about the PAHAL intervention.

## Methods

### Study setting and study population

The city of Mumbai is divided into three large informal geographical regions: the Island City (IS), the eastern suburbs (ES) and the western suburbs (WS). Since PAHAL was to be implemented in all three regions, one slum neighborhood from each region was chosen to serve as a study site. A female health worker was assigned to each slum. This person visited all lanes in the slum during the 1-year data collection period (April 2013 to April 2014). She approached every household in every lane to invite female residents of childbearing age to participate in the study. Women were eligible to participate if they were between the ages of 18 and 45 and were willing to provide oral consent. In households with more than one woman who met this requirement, only one woman was asked to participate. Usually the first woman who met the eligibility criteria was the one selected.

### Data collection

The health workers collected data from study participants using a questionnaire that they administered as an interview in the local language, which is Hindi. The questionnaire was prepared in English and translated into Hindi, then checked using English back-translation. The study was piloted in a community similar to the study communities. Based on the pilot experience, researchers simplified the questionnaire. Several questions were deleted, most relating to risk factors, and other questions were modified. Researchers also made the decision to limit study participation to only one eligible woman from households with more than one member who met study inclusion criteria.

The questionnaire was made up of five sections. Section A was on demography (13 questions); section B, hepatitis B knowledge (4 questions); section C, personal risk related to hepatitis B (7 questions); section D, pregnancy and childbearing history (10 questions); and section E, details of most recent pregnancy (7 questions). With respect to hepatitis B knowledge, a screening question was first asked regarding whether the person had heard about hepatitis B. If the reply was yes, then the hepatitis B knowledge-related questions were asked. If the reply was no, the interviewer proceeded to the next section of the questionnaire. Information about the most recent pregnancy was collected only from women who had given birth within the previous 36 months. If a woman’s youngest child was older than 36 months, the woman was not asked to respond to this section of the questionnaire.

At the time of the interview, study participants’ names and addresses were entered in a register so that implementers of the planned hepatitis B prevention education program would be able to follow up with them at a later date. For data management purposes, each study participant was assigned an area code number and a unique identifying code, both of which were recorded on the paper study interview forms used by the health workers. No personal identifying information was recorded on the interview forms.

### Data analysis

The same data entry operator entered all data from the interview forms into Microsoft Excel. A descriptive data analysis was performed using Microsoft Excel 2007 (Redmond, WA). Simple two-by-two tables were created and respondents’ answers to survey questions were summed and expressed as percentages. For all but one section of the survey, disaggregated findings for the eastern suburbs, western suburbs and Island City were also reported. Responses to questions about hepatitis B knowledge were not disaggregated by geographical area because of the relatively small number of women who reported knowing about hepatitis B.

#### Research ethics

The health workers provided all prospective study participants with information about the study and about the hepatitis B prevention education program that was to be carried out in the community following the study. Before administering the questionnaire, the health workers obtained oral consent from women who agreed to be in the study. No identifying information was collected on the interview form. Following the interview, each study participant was given a leaflet in Hindi about hepatitis B and was invited to participate in the hepatitis B prevention education program that followed. Eligible women who did not want to be in the study were still given leaflets and invited to participate in the program. Since the study posed minimal risk to participants, no ethics committee was asked for formal approval.

## Results

The health workers visited 11,527 households and conducted interviews with 6794 women. Among the completed interview forms submitted by the health workers, 6571 forms were found to be suitable for analysis.

### Demographics of study participants

The participation of women in absolute numbers was lowest from eastern suburbs, followed by the western and then the Island city (ES = 28 %, WS = 34 %, IC = 38 %) (Table 1). Of all the women interviewed, more than 64 % were below the age of 30 years, the percentages being similar across all three areas (ES = 64 %, WS = 65 %, IC = 66 %). The vast majority (89 %) of the women were married (ES = 91 %, WS = 89 %, IC = 87 %); the lowest percentage of married women was from the Island City. A larger proportion (94 %) of women were not employed anywhere (ES = 89 %, WS = 94 %, IC = 96 %); the Island City had the largest percentage of women with no employment. Most women (87 %) reported that their highest level of schooling was below grade 12, with considerable variation between the eastern suburbs cohort and the other two cohorts (ES = 78 %, WS = 92 %, IC = 90 %). More than 20 % of women in the eastern suburbs reported that they had studied in college. A household size of three to five people was the most common; most households (83 %) had five or fewer members. More than a quarter of women from the eastern suburbs reported a household size larger than five, whereas a much smaller percentage reported this household size from the other regions (ES = 28.5 %, WS = 11.7 %, IC = 9.6 %). More than 84 % of households included only one woman aged 18–45. More than one-fifth of women in the eastern suburbs reported having more women of childbearing age in their households, while the other areas had fewer families with more than one woman of childbearing age (ES = 21.4 %, WS = 13.1 %, IC = 11.7 %).

**Table 1:** Study participant demographics *(N*:6571)

	Total		Eastern suburbs	Western suburbs	Island City
	*N = 6571*	Percentage	*N = 2238*	*N = 1808 n(%)*	*N = 2525*
	*n*		*n (%)*		*n (%)*
Age group					
≤20	728	11.1	257 (11.5)	154 (8.5)	317 (12.6)
21–25	1713	26.1	605 (27.0)	436 (24.1)	672 (26.6)
26–30	1807	27.5	616 (27.5)	506 (28.0)	685 (27.1)
31–35	1009	15.4	349 (15.6)	279 (15.4)	381 (15.1)
≥36	1314	20.0	411 (18.4)	433 (23.9)	470 (18.6)
Marital status					
Single	716	10.9	236 (10.6)	153 (8.5)	327 (13.0)
Married	5847	89.0	1999 (89.4)	1652 (91.4)	2196 (87.0)
Divorced	4	0.1	1 (0.0)	1 (0.1)	2 (0.1)
No response	4	0	2 (0.0)	2 (0.1)	0 (0.0)
Employment status					
Employed	417	6.3	232 (10.4)	99 (5.5)	85 (3.4)
Unemployed	6150	93.6	2003 (89.6)	1707 (94.5)	2438 (96.6)
No response	7	0.1	3 (0)	2 (0)	2 (0.1)
Highest level of education					
Did not attend school	576	8.8	254 (11.4)	89 (4.9)	233 (9.2)
Highest level of schooling was below grade 10	2603	39.6	461 (20.6)	1125 (62.3)	1015 (40.2)
Highest level of schooling was below grade 12	2551	38.8	1066 (47.7)	456 (25.2)	1028 (40.7)
Highest level of education was simple graduation or less (equivalent to three years in college)	838	12.8	453 (20.3)	136 (7.5)	249 (9.9)
No response	6	0	4 (0.1)	2 (0.1)	0 (0.0)
Household Size					
≤2	1019	15.5	155 (6.9)	352 (19.5)	511 (20.2)
3–5	4461	67.9	1444 (64.6)	1244 (68.8)	1771 (70.1)
6–8	908	13.8	523 (23.4)	173 (9.6)	212 (8.4)
≥9	183	2.8	114 (5.1)	38 (2.1)	31 (1.2)
No response	3	0	3 (0)	1 (0)	0 (0)
Number of women aged 18–45 in the household					
1	5555	84.6	1756 (78.5)	1571 (86.9)	2228 (88.2)
2	687	10.5	294 (13.1)	170 (9.4)	223 (8.8)
3	257	3.9	148 (6.6)	52 (2.9)	57 (2.3)
4	50	0.8	34 (1.5)	10 (0.6)	6 (0.2)
>4	19	0.3	4 (0.2)	4 (0.2)	11 (0.4)
No response	3	0	2 (0)	1 (0)	0 (0)

### Hepatitis B knowledge

Of all respondents who were asked whether they knew about hepatitis B, only 240 (4 %) replied in the affirmative (ES = 4 %, WS = 8 %, IC = 0.1 %). These 240 women were then asked further about modes of disease transmission, symptoms of acute HBV and conditions caused by chronic HBV (Table 2).

**Table 2:** Hepatitis B knowledge (*N* = 240)

Do you know how hepatitis B spreads?	Yes	No	Do not know
Eating contaminated food^*^	10 %	84 %	6 %
Tattoo or body piercing	6 %	87 %	7 %
Sharing needles or other injection equipment	6 %	86 %	8 %
Transfusion of infected blood	55 %	40 %	4 %
Sexual contact	20 %	73 %	7 %
Mother-to-infant transmission during childbirth	20 %	73 %	6 %
* *All “yes” answers in this section are correct with the exception of this one, for which “no” is the correct answer.*			
Do you know the symptoms of acute hepatitis B?^†^	Yes	No	Do not know
Weakness and fatigue	43 %	53 %	4 %
Loss of appetite	65 %	32 %	3 %
Nausea and vomiting	39 %	55 %	6 %
Upper abdominal discomfort	53 %	42 %	5 %
Tea-coloured urine	46 %	50 %	5 %
Yellowing of skin	43 %	52 %	5 %
† *All “yes” answers in this section are correct.*			
Which of the following conditions may arise from chronic hepatitis B infection?^‡^	Yes	No	Do not know
Liver cirrhosis	14 %	78 %	8 %
Liver cancer	30 %	63 %	7 %
Liver failure	8 %	83 %	10 %
‡ *All “yes” answers in this section are correct.*			
Should a mother with hepatitis B breastfeed her baby?^§^	Yes	No	Do not know
	35 %	60 %	5 %
§ *The correct answer is “yes.”*			

* All “yes” answers in this section are correct with the exception of this one, for which “no” is the correct answer

† All “yes” answers in this section are correct

‡ All “yes” answers in this section are correct

§ The correct answer is “yes”

#### Hepatitis B transmission

Eighty-four percent of respondents knew that eating contaminated food does not cause hepatitis B. Transfusion of blood was identified correctly as a method of acquiring hepatitis B by 55 %. A smaller proportion (22 %) knew that hepatitis B can be transmitted through sexual contact and the same percentage was aware of its spread from a mother to her baby. Only 6 % of respondents knew that hepatitis B can be spread through tattooing or body piercing. The same small proportion of respondents was aware of the potential for hepatitis B to be transmitted by sharing needles or other injection equipment. No respondent correctly answered all six of the questions, and only four respondents correctly answered five of them.

#### Symptoms of hepatitis B

Sixty-five percent of respondents could identify the symptom of loss of appetite correctly, 53 % identified abdominal discomfort correctly, and 46 % knew about tea-colored urine. Less than half (43 %) were aware of the symptoms of weakness and fatigue, and the same percentage knew about yellowing of skin being a symptom in acute cases. Similarly, nausea/vomiting was identified as a symptom by only 39 % of the respondents. Twenty-one respondents (9 %) correctly identified all symptoms.

#### Complications of chronic hepatitis B infection

Thirty percent of respondents were aware that hepatitis B in its chronic phase can cause liver cancer, 14 % knew about liver cirrhosis and 8 % identified liver failure as a complication of the disease. Six respondents (3 %) gave correct answers regarding all three complications.

On being asked if they knew whether a hepatitis B-positive mother should breastfeed her baby, only 35 % of respondents correctly answered in the affirmative, whereas 60 % replied in the negative and 5 % did not know the answer.

#### Hepatitis B and most recent pregnancy

Only women whose youngest child was no older than 36 months were asked questions about their most recent pregnancy. A total of 739 (11.2 %) women met this condition (Table 3).

**Table 3:** Pregnancy behavior among women^a^ (*N* = 739)

	Total number *n* (%)	Eastern suburbs *n* (%)	Western suburbs *n* (%)	Island City *n* (%)
Age of most recent child				
12 months or less	440 (60)	144 (62)	168 (60)	128 (56)
13–24 months	281 (38)	87 (37)	110 (40)	84 (37)
25–36 months	18 (2)	3 (1)	0 (0)	15 (7)
Tested for hepatitis B during pregnancy?				
Yes	162 (22)	43 (18)	103 (37)	16 (7)
No	267 (36)	165 (71)	73 (26)	29 (13)
No reply	310 (42)	26 (11)	102 (37)	182 (80)
Hepatitis B status of those tested (*N* = 162)				
Positive	2 (1)	2 (5)	0	0
Negative	158 (98)	41 (95)	103 (100)	14 (88)
Not known	2 (1)	0	0	2 (12)

^a^Women who reported giving birth within the previous 36 months

More than half of the 739 women had given birth to their last child in the previous year, while mothers of children older than age two formed a very small percentage of the cohort (2 %). Twenty-two percent of women reported that they had taken a hepatitis B test during pregnancy, with responses varying considerably across the three study areas (ES = 18 %, WS = 37 %, IC = 7 %). Among women who reported being tested for hepatitis B, two indicated that they were hepatitis B-positive.

A large majority of children (81 %) had been delivered in the city of Mumbai, with the highest percentage from Island City (ES = 71 %, WS = 84 %, IC = 89 %) (Table 4). Eighty-one percent of women reported that their children had received the first dose of the hepatitis B vaccine before leaving the hospital as newborns. Island City stood apart from the other two study sites in regard to this question, with only 56 % of Island City women reporting administration of the first dose, while 44 % did not know what their child’s vaccination status had been upon leaving the hospital. Seventy percent of children went on to receive three complete doses of the vaccine, according to their mothers’ reports. The eastern suburbs had the highest level of vaccination, and Island City, the lowest (ES = 84 %, WS = 74 %, IC = 52 %). A large proportion (43 %) of Island City mothers were unaware of the current vaccination state of their children.

**Table 4:** Hepatitis B vaccination status of children^a^ as reported by their mothers (*N* = 739)

	Total number *n* (%)	Eastern suburbs *n* (%)	Western suburbs *n* (%)	Island City *n* (%)
Place of delivery				
Mumbai	602 (81)	165 (71)	234 (84)	203 (89)
Outside	103 (14)	61 (26)	22 (8)	20 (9)
No answer	34 (5)	8 (3)	22 (8)	4 (2)
Newborn hepatitis B vaccination status upon leaving hospital				
Vaccinated before leaving hospital	599 (81)	216 (92)	257 (92)	126 (56)
Not vaccinated before leaving hospital	4 (1)	4 (2)	0 (0)	0 (0)
Do not know	119 (16)	9 (4)	10 (4)	100 (44)
No reply	17 (2)	5 (2)	11 (4)	1 (0)
Child’s current hepatitis B vaccination status				
Three complete doses	520 (70)	197 (84)	206 (74)	117 (52)
Partially vaccinated	80 (11)	18 (8)	51 (18)	11 (5)
Do not know	122 (17)	14 (6)	10 (4)	98 (43)
No reply	17 (2)	5 (2)	11 (4)	1 (0)

^a^Children born within the previous 36 months

## Discussion

The present study was conducted to assess HBV knowledge levels among women of childbearing age in three Mumbai slums in order to help determine how their HBV-related needs should be addressed through a prevention education program. Among 6571 study participants who were asked if they knew about this disease, less than five percent answered yes. The 240 women in this subset demonstrated high levels of correct knowledge about some modes of transmission and disease symptoms, but low levels of correct knowledge about other modes of transmission and disease symptoms. Small proportions of this subset knew that chronic hepatitis B may cause liver cancer, liver cirrhosis and liver failure. Less than one-quarter of 739 study participants with children aged 36 months or younger reported undergoing a hepatitis B test during pregnancy. On the other hand, more than two-thirds of women indicated that their children had received the recommended three doses of HBV vaccine.

Other studies conducted in various populations elsewhere in India and around the world have revealed less than optimal awareness of hepatitis B and the presence of misconceptions about the disease [10–14]. For example, a study conducted among 430 women of childbearing age in rural Pakistan reported that less than half of women had correct knowledge about HBV vaccination, with especially poor knowledge among women of lower socioeconomic status [14]. Our study results further substantiate concerns about a lack of public awareness and knowledge being a major driver of ongoing mother-to-child transmission of HBV globally, while at the same time indicating a need for setting-specific interventions in Mumbai. Consideration should be given to investigating reasons for the lack of knowledge about HBV within our study communities, including whether different health care providers might have different approaches to communicating with women about how to protect themselves and their children from HBV.

There was more correct knowledge about some modes of HBV transmission than others in our study population. Only one-fifth of women knew about sexual contact as a mode of transmission, and the same proportion knew that infants could acquire HBV from infected mothers during childbirth. Also important from a program planning perspective was the finding that more than half of women wrongly thought that a mother with hepatitis B should not breastfeed her baby. Given the poor breastfeeding numbers prevalent in urban areas, especially Mumbai [15], inaccurate beliefs of this nature are potentially harmful to an already at-risk newborn's health. HBV prevention education thus also serves as an opportunity to contribute to the broader child health agenda.

Our study findings raised additional concerns about the nature of the HBV prevention efforts in India. Only 22 % of study participants with children aged 36 months or younger indicated that they had been tested for hepatitis B during pregnancy. Seventy percent of the same subset of women indicated that their children had received three doses of hepatitis B vaccine, but reported vaccination levels varied greatly across the three study areas. The World Health Organization recommends administering the first dose of HBV vaccine to all newborns within 24 h of birth regardless of their mothers’ HBV status [16]. Furthermore, the likelihood of the hepatitis B vaccine schedule being completed—along with other immunizations—is found to improve when vaccination is initiated at birth [17]. Study results thus suggest that there is considerable work to be done in terms of making women more aware of testing procedures and the implications of the results, along with accepted recommendations for vaccination. This is particularly true in Island City, where the largest proportion (56 %) of mothers with children under 12 months reside. At the same time, other types of research are needed to acquire insight into the roles of health service providers in facilitating or hindering the uptake of HBV screening and prevention recommendations by women during pregnancy and delivery and afterwards.

### Study limitations

The survey was conducted for the express purpose of determining community needs in relation to a planned HBV education intervention and also to gauge the size of the potential target audience for the intervention. It was not designed with scientific publishing in mind. No systematic sampling was carried out, as the mandate was to include all willing subjects in the target group. Although care was taken to train the health workers in administering the survey questions, there may have been some observer bias. Additionally, since the survey was administered over a year-long period, there may have been some information transfer from the women surveyed earlier to those surveyed later. The study was a simple survey to assess knowledge levels. It did not include a data analysis plan to test for statistical significance; the absolute information obtained was used to inform the activities of the project. Although sociodemographic information was collected, survey findings were not analyzed on the basis of age or education levels. Further research may be able to throw light on the relevance of these variables in designing HBV education interventions.

The information obtained regarding whether study participants were tested for hepatitis B during pregnancy was not, in most cases, supported by actual evidence in the form of the test reports. Similarly, in the absence of vaccination cards for verification, information on the percentage of childhood vaccinations administered was also as per the mother's memory and comprehension. The likelihood of misreporting may have been reduced because interviewers sought to help study respondents avoid confusing the hepatitis B vaccine and the bacille Calmette-Guerin vaccine by describing the injection site (the left lateral thigh, for the former, or the left deltoid, for the latter).

## Conclusion

Despite the availability of a safe HBV vaccine that is free for newborns and infants in many parts of India, preventing mother-to-child transmission of HBV remains a major challenge. Low awareness of HBV among women of childbearing age in Mumbai-area slums indicates a need for educational interventions targeting this population. Additional research is needed to further elucidate the barriers to implementing key HBV control measures, such as screening pregnant women and achieving universal HBV vaccination coverage, including administration of the first dose of vaccine within 24 h of birth.

## Abbreviations

ES: eastern suburbs
IC: Island City
HBsAg: hepatitis B surface antigen
HBV: hepatitis B virus
UNICEF: The United Nations Children's Fund
WHO: World Health Organization
WS: western suburbs
